# Cytogenetic study on antlions (Neuroptera, Myrmeleontidae): first data on telomere structure and rDNA location

**DOI:** 10.3897/CompCytogen.v10i4.10775

**Published:** 2016-11-25

**Authors:** Valentina G. Kuznetsova, Gadzhimurad N. Khabiev, Boris A. Anokhin

**Affiliations:** 1 Zoological Institute, Russian Academy of Sciences, Universitetskaya nab. 1, 199034, St. Petersburg, Russia; 2 Prikaspiyskiy Institute of Biological Resources, Dagestan Scientific Centre, Russian Academy of Sciences, ul. M. Gadzhieva 45, 367025 Makhachkala, Russia

**Keywords:** TTAGG, rDNA, fluorescence *in situ* hybridization, Palpares libelluloides, Acanthaclisis occitanica, Distoleon tetragrammicus, Libelloides macaronius, Myrmeleontidae, Ascalaphidae, Neuroptera

## Abstract

Myrmeleontidae, commonly known as “antlions”, are the most diverse family of the insect order Neuroptera, with over 1700 described species (in 191 genera) of which 37 species (in 21 genera) have so far been studied in respect to standard karyotypes. In the present paper we provide first data on the occurrence of the “insect-type” telomeric repeat (TTAGG)*_n_* and location of 18S rDNA clusters in the antlion karyotypes studied using fluorescence *in situ* hybridization (FISH). We show that males of *Palpares libelluloides* (Linnaeus, 1764) (Palparinae), *Acanthaclisis occitanica* (Villers, 1789) (Acanthaclisinae) and *Distoleon tetragrammicus* (Fabricius, 1798) (Nemoleontinae) have rDNA clusters on a large bivalent, two last species having an additional rDNA cluster on one of the sex chromosomes, most probably the X. (TTAGG)*_n_* - containing telomeres are clearly characteristic of *Palpares libelluloides* and *Acanthaclisis occitanica*; the presence of this telomeric motif in *Distoleon tetragrammicus* is questionable. In addition, we detected the presence of the (TTAGG)*_n_* telomeric repeat in *Libelloides macaronius* (Scopoli, 1763) from the family Ascalaphidae (owlflies), a sister group to the Myrmeleontidae. We presume that the “insect” motif (TTAGG)*_n_* was present in a common ancestor of the families Ascalaphidae and Myrmeleontidae within the neuropteran suborder Myrmeleontiformia.

## Introduction

The ends of eukaryotic chromosomes are capped with complex nucleoprotein structures, the telomeres, which preclude fusion, recombination and degradation of the chromosome ends and thus maintain the genome integrity. In a large number of organisms, telomeric DNA consists of highly repetitive short sequences. Although telomeres are diversified in their DNA sequence composition in different eukaryotic groups, several phylogenetic lineages demonstrate highly conserved motifs. In animals, three main types of telomeric repeats are known: TTAGGG, TTAGGC, and TTAGG. Motif (TTAGGG)*_n_* prevails in the multicellular animals, except roundworms and arthropods, and is probably ancestral for all Metazoa; motif (TTAGGC)*_n_* is characteristic of nematodes; motif (TTAGG)*_n_*, which is a derivative of (TTAGGG)*_n_*, occurs in all arthropod groups (sea spiders, chelicerates, myriapods, crustaceans, and hexapods), supporting their origin from a common ancestor (Traut et al. 2007, Gomes et al. 2010). The (TTAGG)*_n_* telomeric motif is commonly found among different higher insect taxa, and this telomere structure is supposed to be phylogenetically ancestral in the class Insecta in general (Frydrychová et al. 2004). However, the insect-type consensus telomeric sequence is known to be lost independently during insect evolution (Frydrychová et al. 2004, Lukhtanov and Kuznetsova 2010, Gokhman et al. 2014). Further on, the loss and subsequent gain of typical telomeres may occur repeatedly as it has been indicated in Coleoptera (Frydrychová and Marec 2002) and recently in Heteroptera (Pita et al. 2016). However, it is worth noting that the number of species with known telomere structure is extremely low in each insect order, including Neuroptera (Frydrychová et al. 2004).

Neuroptera, also known as Planipennia, are a highly heterogeneous insect order, with 5803 species described in 16 families (Oswald 2016). In this group, telomere structure has so far been described in only two species, *Protidricerus japonicus* (McLachlan, 1891) from the family Ascalaphidae (Okazaki et al. 1993) and *Chrysoperla carnea* (Stephens, 1836) from the family Chrysopidae (Frydrychová et al. 2004). The former species was found to have the insect-type telomeric motif (TTAGG)*_n_*, whereas the latter species was reported to lack this motif. Considering the heterogeneity in the presence of the TTAGG telomeric repeats discovered in Neuroptera, it thus could be interesting to verify the presence of this motif in other members of the order.

Within Neuroptera, the family Myrmeleontidae, commonly known as “antlions”, due to the fiercely predatory habits of their larvae, is the most diverse group having worldwide distribution. This family is considered monophyletic, with over 1700 extant species in 191 genera. In Myrmeleontidae, as many as 12 subfamilies, among them Palparinae, Pseudimarinae, Stilbopteryginae, Dimarinae, Echthromyrmicinae, Dendroleontinae, Nemoleontinae, Glenurinae, Myrmecaelurinae, Acanthaclisinae, Brachynemurinae, and Myrmeleontinae (Krivokhatsky 2011), but most commonly only three, Stilbopteryginae, Palparinae and Myrmeleontinae (Stange 2004, Badano et al. 2016), are recognized. Myrmeleontidae, together with the families Psychopsidae, Nemopteridae, Nymphidae and Ascalaphidae, form the monophyletic suborder Myrmeleontiformia (= the superfamily Myrmeleontoidea) that is a derived lineage of Neuroptera diversified in the Jurassic period (Badano et al. 2016). Different phylogenetic analyses based on morphological and genetic data established a sister-group relationship between Myrmeleontidae and Ascalaphidae (Badano et al. 2016). Ascalaphidae, or owlflies, are a smaller family, with about 430 described species in 100 genera distributed in all the biogeographic regions (Tjeder 1992). The family is subdivided into two main subfamilies, Haplogleniinae and Ascalaphinae (Henry 1978).

Until now, the cytogenetic studies in the Myrmeleontidae have been carried out on 37 species from 21 genera, and were focused exclusively on the basic features of the karyotypes such as chromosome numbers and sex determination systems (reviewed in Kuznetsova et al. 2015).

The aim of the present study is to further characterize chromosomes of antlions and to study their evolution by exploring the telomere structure and chromosomal location of the major ribosomal RNA (rRNA) genes using fluorescence *in situ* hybridization (FISH). The FISH technique was applied for the first time in the family Myrmeleontidae.

We examined the presence/absence of TTAGG telomeric repeats and location of the rDNA clusters in *Palpares libelluloides* (Linnaeus, 1764), *Distoleon tetragrammicus* (Fabricius, 1798) and *Acanthaclisis occitanica* (Villers, 1789) from the family Myrmeleontidae. In addition, we studied telomere structure in *Libelloides macaronius* (Scopoli, 1763) belonging to the sister family Ascalaphidae.

## Material and methods

### Material

Three antlion species, involving three different genera from three subfamilies (*sensu* Krivokhatsky 2011), i.e. Palparinae (*Palpares libelluloides*), Nemoleontinae (*Distoleon tetragrammicus*) and Acanthaclisinae (*Acanthaclisis occitanica*), as well as the only owlfly species from the subfamily Ascalaphinae (*Libelloides macaronius*) were studied. The specimens were collected by G. Khabiev from May to October 2015 in the Republic of Dagestan (North-East Caucasus, Russia). In the field, adult individuals were fixed in a solution of 96% alcohol and glacial acetic acid (3:1) and then stored at 4°C until required. Collection localities and chromosomal traits of each species are given in Table 1.

**Table 1.** Examined material and main karyotypic features obtained during the present study.

Taxon	Sampling locality and month and year of collection	Number of studied males	Diploid karyotype	Telomeric sequence	18S rDNA clusters location
Myrmeleontidae					
**Palparinae**					
*Palpares libelluloides* (Linnaeus, 1764)	Russia, Dagestan, near Makhachkala 42°59'59.6"N 47°13'33.0"E, VI.2015	2	24 + XY	(TTAGG)*_n_*	AA*
**Nemoleontinae**					
*Distoleon tetragrammicus* (Fabricius, 1798)	Russia, Dagestan, near Makhachkala 43°00'28.7"N 47°14'51.3"E, VII.2015	2	14 + XY	?**	AA + X
**Acanthaclisinae**					
*Acanthaclisis occitanica* (Villers, 1789)	Russia, Dagestan, near Makhachkala 43°00'28.7"N 47°14'51.3"E, VII.2015	2	16 + XY	(TTAGG)*_n_*	AA + X
Ascalaphidae					
**Ascalaphinae**					
*Libelloides macaronius* (Scopoli, 1763)	Russia, Dagestan, near Gelinbatan village 41°56"50"N, 48°10"2"E, VII.2015	1	-***	(TTAGG)*_n_*	-***

*A pair of autosomes; ** Ambiguous data; *** Missing data

### Telomere and rDNA detection by FISH

Chromosome preparations were obtained from male gonads. Testes were dissected in a drop of 45% acetic acid and squashed. The coverslips were removed using dry ice. Prior to FISH treatment, the preparations were examined using phase contrast microscopy.

FISH with (TTAGG)*_n_* and 18S rDNA probes was applied as previously reported for some other insects (Kuznetsova et al. 2015, Maryańska-Nadachowska et al. 2016, Golub et al. 2016). In brief, chromosome preparations were treated with 100 µg/ml RNase A and 5 mg/ml Pepsin solution to remove excess RNA and proteins. Chromosomes were denatured on a slide in a hybridization mixture with biotinylated 18S rDNA probe from the genomic DNA of *Pyrrhocoris apterus* (Linnaeus, 1758) and rhodaminated (TTAGG)*_n_* probe with addition of salmon sperm DNA and then hybridized for 36 h. Hybridization signals were detected with NeutrAvidin-FITC.

Chromosomes were mounted in antifade medium (ProLong Gold antifade reagent with DAPI; Invitrogen) and covered with a glass coverslip. Chromosome slides were analyzed under a Leica DM 6000 B microscope. Images were taken with a Leica DFC 345 FX camera using Leica Application Suite 3.7 software with an Image Overlay module.

## Results

**Figures 1–10.**
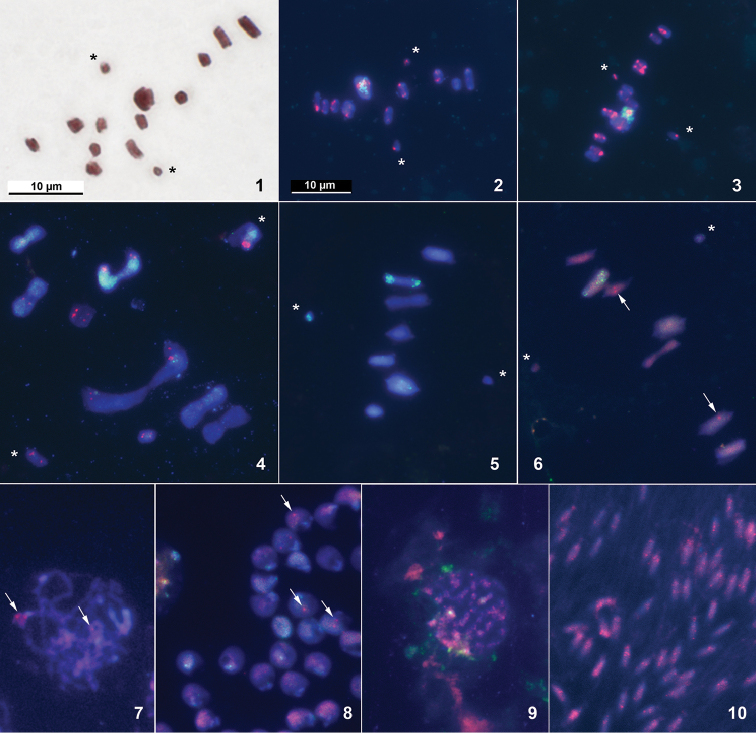
Different stages of spermatogenesis in antlion species *Palpares libelluloides* (**1–3**), *Acanthaclisis occitanica* (**4**), *Distoleon tetragrammicus* (**5–8**) and owlfly species *Libelloides macaronius* (**9–10**) after standard staining (**1**) and FISH with the 18S rDNA and telomeric (TTAGG)*_n_* probes (**2–10**). **1–3** MI, n = 12 + X + Y **4** diakinesis/MI transition, n = 8 + X + Y **5, 6** MI, n = 7 + X + Y **7** pachytene **8** spermatids **9** mitotic metaphase **10** spermatids. Asterisks mark sex chromosomes; red signals identify the (TTAGG)*_n_* - positive telomeres (arrowed); green signals identify rDNA clusters. Scale bar on Fig. **2** also applies to Figs **3–10**.

In male *Palpares libelluloides*, we found 12 autosomal bivalents and X and Y univalent chromosomes (Fig. 1), confirming the chromosome number, 2n = 26 (24 + XY), reported by Kuznetsova et al. (2015). FISH with “insect” telomeric probe (TTAGG)*_n_* produced strong hybridization signals on the chromosome ends at metaphase I (Figs 2, 3) and other stages of meiosis (not shown). Some differences in hybridization intensity could be seen among different bivalents and between homologous telomeres. The rDNA probe detected 18S rDNA clusters on a large pair of autosomes (Figs 2, 3).

In male *Acanthaclisis occitanica*, we found 8 bivalents of various sizes and the X and Y univalent chromosomes, suggesting 2n = 18 (16 + XY). This species is cytogenetically studied for the first time. Its karyotype includes one exceptionally large pair of bi-armed autosomes, another pair of large bi-armed autosomes and the two sex chromosomes, with the longer metacentric chromosome presumably being the X, while the other metacentric chromosome − the Y; the detailed morphology of other chromosomes remained unknown. The majority of bivalents and sex chromosomes displayed strong signals of the telomere probe. As for the heterogeneity for presence/absence and intensity of the (TTAGG)*_n_* signals, it could be explained by technical artifacts. Apart from a few scattered signals, the ribosomal probe detected a considerable accumulation of the 18S rDNA sequence on the second large pair of bi-armed autosomes and, additionally, on the putative X chromosome (Fig. 4).

In male *Distoleon tetragrammicus*, we found 7 autosomal bivalents and X and Y univalent chromosomes (Figs 5, 6). An additional univalent, most likely a B-chromosome, which precociously segregates together with one of the sex chromosomes, was occasionally observed in first metaphase nuclei (not shown). The data obtained suggest a diploid karyotype of 2n = 16 (14 + XY) in contrast to 2n = 18 (16 + XY) reported by Kuznetsova et al. (2015). FISH with (TTAGG)*_n_* probe has detected no hybridization signals in the first metaphase nuclei (Fig. 5). However, weak and scarce (TTAGG)*_n_* hybridization signals of uncertain location could be seen in separate chromosomes of different stages as well as in the spermatids (Figs 6–8). The rDNA probe revealed 18S rDNA clusters both on a large pair of autosomes (possibly the third pair), and one of the sex chromosomes (Figs 5, 6).

In male *Libelloides macaronius*, the karyotype remained unknown. However the (TTAGG)*_n_* -positive signals could be clearly observed in some cells including spermatids (Figs 9, 10).

## Discussion

### Karyotypes

As summarized recently (Kuznetsova et al. 2015), the karyotypes are currently known for 37 species of the Myrmeleontidae representing about 2.2% of the extant antlion species. The studied species belong to 21 genera and 9 subfamilies (*sensu* Krivokhatsky 2011) and were shown to have an XY-sex chromosome system as well as diploid chromosome numbers ranging from 14 to 26. The highest numbers, 2n = 22, 24 and 26, occur only in a more basal subfamily Palparinae, whereas lower numbers, 2n = 14, 16 and 18, are encountered in other subfamilies. Since the sister group to the Myrmeleontidae, i.e. the family Ascalaphidae, is characterized by higher chromosome numbers, usually 2n = 22, a higher number was suggested to be ancestral for antlions (Kuznetsova et al. 2015). The karyotypes of 2n = 16 and 2n = 18, which we recently found in *Distoleon tetragrammicus* (Nemoleontinae) and *Acanthaclisis occitanica* (Acanthaclisinae) respectively, corroborate this assumption. These karyotypes are further discussed below.

In our previous paper (Kuznetsova et al. 2015), *Distoleon tetragrammicus* was reported to have 2n = 18, i.e., having two additional pairs of autosomes. A plausible explanation for this disagreement is that a univalent displaced from the equatorial plane was erroneously identified as a bivalent at the first metaphase in the only studied male (see Fig. 2 in Kuznetsova et al. 2015). It is worth noting that a univalent segregating precociously with the sex chromosomes at the first metaphase was also observed in some nuclei of male *Distoleon tetragrammicus* during the present study (see Results). We suggest that these univalent chromosomes are additional or so-called B chromosomes. One or two additional chromosomes that do not belong to the regular karyotype and are similar in their meiotic behavior to the sex chromosomes were repeatedly observed in different neuropteran species, including the antlion *Myrmeleon mexicanus* Banks, 1903 (Hughes-Schrader 1983). The effect of the supernumeraries varies in different species. For example, in *Hemerobius marginatus* Stephens, 1836 from another neuropteran family Hemerobidae, additional chromosomes influence the segregation of the sex chromosomes in meiosis (Nokkala 1986). The problem of B chromosomes in antlions needs to be further addressed in the future.

The karyotype of *Acanthaclisis occitanica* differs by having both very large autosomal bivalent and sex chromosome (supposedly the X). To our knowledge, this karyotype structure was never reported for the Myrmeleontidae. However, it is worth noting that antlion karyotypes were almost exclusively illustrated with drawings in the past (as opposed to photos) with no significant details of the chromosome structure and size reported.

### Telomeres

The data obtained in the present study demonstrate for the first time the presence of the insect-type telomeric repeat (TTAGG)*_n_* in antlions. We have reliably shown that this motif is characteristic of *Palpares libelluloides* and *Acanthaclisis occitanica*. The third examined antlion species, *Distoleon tetragrammicus*, in which only rare TTAGG-positive signals of uncertain location were detected at best, most likely does not have the canonical (TTAGG)*_n_* insect telomeric motif. However, the TTAGG sequence could actually be present in the telomeres but in very low copy numbers, making it difficult to detect this sequence by FISH. Consequently, we consider the data on *Distoleon tetragrammicus* as preliminary and therefore deserving further clarification. We also showed that the (TTAGG)*_n_* repeat was present in telomeres of *Libelloides macaronius* (Ascalaphidae). Earlier, this telomeric motif was recorded for another owlfly species, *Protidricerus japonicas*, by Frydrychová et al. (2004). Together with the results on *Protidricerus japonicus*, our data suggest that the (TTAGG)*_n_* telomere sequence found in species of Ascalaphidae and Myrmeleontidae was characteristic of the common ancestor of these sister families. The detection of this repeat in the most basal antlion subfamily examined so far, the Palparinae (*Palpares libelluloides*), further corroborates this suggestion.

At present, the only other neuropteran species with known telomere structure is *Chrysoperla carnea* *s. lato* belonging to the large worldwide family Chrysopidae (green lacewings). Based on the Southern hybridization results, Frydrychová et al. (2004) have shown that *Chrysoperla carnea* is (TTAGG)*_n_* -negative. Despite the relatively small size of the order Neuroptera including only 5803 extant species (Oswald 2016), the data on telomere structure are still highly insufficient, and further studies are needed to fully understand the organization of telomeres in different families of this insect order.

### Ribosomal clusters

Ribosomal gene markers have provided useful information regarding chromosome evolution in different groups of insects. In some groups, the number and chromosomal localization of rDNA clusters, usually located in the nucleolus organizing regions (NORs), serve as additional markers to characterize species and higher taxa (Nguyen et al. 2010, Gokhman et al. 2014), whereas in other groups they currently are the only available cytogenetic markers to differentiate species with similar karyotypes (Golub et al. 2016). Our data represent the first mapping experiments for the major rRNA genes (i.e. genes for 18S, 5.8S and 28S rRNA) not only in Myrmeleontidae but also in Neuroptera in general. The three species studied, *Palpares libelulloides*, *Acanthaclisis occitanica* and *Distoleon tetragrammicus*, showed the occurrence of one (in the first species) or two (in the two last species) rDNA clusters in their haploid karyotypes. In each species, these clusters are located on both autosomes of a particular large pair. In *Acanthaclisis occitanica* and *Distoleon tetragrammicus*, another rDNA site is present on one of the sex chromosomes. Although it is at present impossible to identify homeologous chromosomes between different neuropteran species, we can suggest a single chromosome pair carrying major rDNA clusters as an ancestral state in antlions.

## Conclusions

Our study contributes to the current knowledge of cytogenetics of the neuropteran family Myrmeleontidae. The principal outcomes of this study are: (1) the discovery of one or two major rDNA clusters per haploid karyotype; the clusters are located either only on a pair of autosomes in a particular species or, additionally, on one of the sex chromosomes in another two studied species and (2) the discovery of the insect-type (TTAGG)*_n_* telomeric sequence at least in two of the three studied species. Because the (TTAGG)*_n_* sequence is likewise found in the two studied owlfly species, we suggest that this telomere structure was inherent in the last common ancestor of the phylogenetic lineage Myrmeleontidae + Ascalaphidae.
